# Description and Control Levels of Hypertriglyceridemia Among Patients Attending Family Medicine Clinics in Riyadh, Saudi Arabia

**DOI:** 10.7759/cureus.94562

**Published:** 2025-10-14

**Authors:** Saeed Alqahtani, Meshal Alhadlaq, Hamad Alkanhal

**Affiliations:** 1 Department of Aviation Medicine, King Abdulaziz Medical City (KAMC) Ministry of National Guard Health Affairs, Riyadh, SAU; 2 Department of Family Medicine, King Abdulaziz Medical City (KAMC), Riyadh, SAU; 3 Department of Family Medicine, King Abdulaziz Medical City (KAMC) Ministry of National Guard Health Affairs, Riyadh, SAU

**Keywords:** dyslipidemia, hyperlipidemia, hypertriglyceridemia, level of control, triglycerides (tg)

## Abstract

Background

Hypertriglyceridemia (HTG) is a prevalent lipid disorder associated with an increased risk of cardiovascular disease (CVD) and acute pancreatitis. Effective management of HTG remains a significant public health concern, particularly in Saudi Arabia, where prevalence rates vary widely. This study aims to assess the level of control of HTG among patients attending family medicine clinics in Riyadh, Saudi Arabia, and to evaluate the effectiveness of different treatment modalities.

Methods

A cross-sectional chart review was conducted on patients from the National Guard Health Affairs Primary Health Care Centers in Riyadh between January 1, 2019, and December 31, 2022. Adults (≥18 years) with triglyceride levels ≥1.7 mmol/L and at least two visits during the study period were included. Patients with renal or liver disease and certain metabolic conditions were excluded. Data were extracted from electronic medical records, including demographics, comorbidities, lipid profiles, and treatment regimens. Statistical analysis was performed to assess triglyceride level reductions and treatment efficacy.

Results

Among 415 patients (median age 62 years), 245 (59%) were female and 170 (41%) were male. Triglyceride levels significantly decreased from a median of 2.90 mmol/L to 1.70 mmol/L post-treatment (p < 0.001). Statins, particularly simvastatin 10 mg, showed a significant effect on triglyceride reduction (p = 0.049). Lifestyle modifications and marital status were also associated with improved outcomes.

Conclusion

HTG management in primary care settings showed promising results, with significant triglyceride reductions. Further studies should explore adherence to lifestyle interventions and additional pharmacological options to enhance HTG control and reduce CVD risk.

## Introduction

Hypertriglyceridemia (HTG) is a condition commonly defined and characterized by fasting serum triglyceride concentrations of 150 mg/dL (1.7 mmol/L) or above [1]. Although there is no apparent global consensus on HTG classification, according to the 2018 American College of Cardiology/American Heart Association (ACC/AHA) guidelines, moderate HTG is defined as 150-499 mg/dL (1.7-5.6 mmol/L), and severe HTG is defined as 500 mg/dL (5.65 mmol/L) or more [2]. It can be part of a genetically acquired disorder or secondary to certain conditions such as obesity, type 2 diabetes mellitus, or the use of certain drugs [2].

HTG is considered one of the most common forms of dyslipidemia [3]. A United States study published in 2020 examined data from 2007 to 2014 and estimated that the total prevalence of HTG in the general population was around 25.9% [4]. Furthermore, the same study indicated that male individuals had a higher prevalence compared to females (28.7% and 21.5%, respectively) [4]. A Spanish study conducted in 2020 estimated the prevalence of HTG in the global population to be around 27.0% [5]. Agreeing with the previous study, male individuals were more prevalent than females (34.6% and 21.4%, respectively) [5]. Locally, a study that examined both rural and urban Saudis in 2008 estimated the prevalence of HTG in Saudi Arabia to be around 40.3% [6]. In addition, a more recent local study published in 2018 at a Saudi university clinic in Al Hofuf indicated that the prevalence of HTG was around 17% [7].

Previously, the association between HTG and cardiovascular disease (CVD) was debatable; however, recent studies have demonstrated that specific subcategories of HTG are associated with increased cardiovascular risk [8-9]. Moreover, severely elevated triglyceride levels have been proven to induce acute pancreatitis [8-9]. Unfortunately, HTG is an asymptomatic condition and is usually discovered accidentally or during routine screening [1]. Therefore, management mainly focuses on reducing the incidence of these complications.

Different treatment regimens are involved in lowering TG levels depending on HTG etiology. These include non-pharmacological approaches such as lifestyle modifications (decreasing alcohol intake, tobacco use cessation, increasing physical activity), dietary modification, and promotion of weight loss [10-11]. Pharmacological approaches include the administration of fibrates, omega-3 fatty acids, and statins [10-11].

Therefore, the main aim of this study is to determine the level of control of HTG among patients attending Family Medicine Clinics of the National Guard Health Affairs in Riyadh, Saudi Arabia, while also comparing different treatment regimens used in HTG.

## Materials and methods

This cross-sectional chart review study was conducted involving patients in the National Guard Health Affairs Primary Health Care Centers, including the Health Care Specialty Center (HCSC), King Abdulaziz City Housing (Iskan), and the National Guard Comprehensive Specialized Clinic (NGCSC) in Riyadh, Saudi Arabia, during the period from January 1, 2019, until December 31, 2022. All adults (≥18 years old), both males and females, who had triglyceride (TG) levels of ≥1.7 mmol/L and had a minimum of two visits between January 1, 2019, and December 31, 2022, were included in the study.

The exclusion criteria included any individuals with the following diagnoses: any renal-related disease such as proteinuria, uremia, glomerulonephritis, nephrotic syndrome, or post-renal transplant; liver-related diseases such as liver cirrhosis, hepatitis, liver carcinoma or neoplasms, alcoholic liver disease, or chronic liver disease. Furthermore, patients diagnosed with hypothyroidism, multiple myeloma, or systemic lupus erythematosus were also excluded from the study.

The recommended sample size was 377, calculated using the Raosoft website (http://www.raosoft.com/samplesize.html) with a confidence level of 95% and a margin of error of 5%. The sample size was eventually increased to 415 to compensate for incomplete or missing data. Simple random sampling was performed using the R statistical software (‘sample’ function) from the list of all eligible patients who met the inclusion and exclusion criteria. This approach was chosen to ensure that every patient had an equal probability of selection, thereby minimizing selection bias and improving the representativeness of the final sample. 

Patients’ documents and electronic files were accessed using the electronic medical record system of the National Guard Health Affairs facilities (BESTCare). Data were collected and cleaned by two researchers in a password-protected Microsoft Excel sheet after obtaining approval from the IRB of King Abdullah International Medical Research Center (KAIMRC) (Approval #IRB/0498/23) and were subsequently imported into R software (version 4.2.2).

The extracted data included demographic information (age, gender, and BMI), comorbidities, number of visits to Family Medicine clinics, first abnormal laboratory results during the selected period, and the latest laboratory results (lipid profile in mmol/L, including low-density lipoprotein cholesterol (LDL-C), high-density lipoprotein cholesterol (HDL-C), TG, and total cholesterol (TC). Data also included details on current HTG treatments (type of medication, date of initiation, dose at initiation, and latest dose).

To elaborate on the sampling process, the three primary health care (PHCs) were identified, and all patients with abnormal TG results (≥1.7 mmol/L) were extracted, totaling 24,832 patients. Next, 5,519 patients were excluded from the study as they had only one visit. Therefore, the total number of patients after applying the inclusion criterion of two or more visits during the period from January 1, 2019, to December 31, 2022, was 19,313. Afterwards, 4,969 patients were excluded based on the exclusion criteria. The final total number of patients after applying both the inclusion and exclusion criteria was 14,344.

The normality of the data was assessed using a histogram and the Shapiro-Wilk test. Continuous variables were presented as medians and interquartile ranges, while categorical data were presented as numbers and percentages. The Wilcoxon rank-sum test was used to assess the association between differences in TG levels, demographic characteristics, and medications used. A p-value of less than 0.05 was considered statistically significant.

## Results

The study included 415 individuals, with a median age of 62 years (IQR 58-69). The age distribution included 8.9% aged 38-48 years, 24% aged 49-59 years, 49% aged 60-70 years, and 18% aged 71 years or older. Of these, 245 (59%) were female and 170 (41%) were male. A total of 360 (87%) were married, 32 (7.7%) widowed, 13 (3.1%) single, and 10 (2.4%) divorced. Almost all participants were Saudi nationals (99%). Hospital distribution showed that 31 (7.5%) were from Um Alhamam Clinic, 60 (14%) from Iskan Clinic, and 324 (78%) from Khashm Alaan Clinic.

The BMI of participants was as follows: 278 (67%) were obese, 110 (27%) were overweight, and 27 (6.5%) had a healthy weight. Comorbidities included diabetes mellitus in 152 (37%), dyslipidemia in 136 (33%), hypertension in 57 (14%), osteoarthritis in 19 (4.6%), vitamin D deficiency in 17 (4.1%), cataracts in 15 (3.6%), and UTIs in 10 (2.4%). Routine check-ups and follow-ups were reported by 57 (14%) and 41 (9.9%) participants, respectively. All demographic characteristics of participants are included in Table 1.

**Table 1:** Demographic characteristics of study participants. ^1^Data are presented as n (%); median (IQR).

Characteristics	N = 415^1^
Age	62 (58-69)
38-48 years	37 (8.9%)
49-59 years	99 (24%)
60-70 years	203 (49%)
71 years and above	76 (18%)
Gender	
Female	245 (59%)
Male	170 (41%)
Marital status	
Divorced	10 (2.4%)
Married	360 (87%)
Single	13 (3.1%)
Widowed	32 (7.7%)
Nationality	
Non-Saudi	4 (1.0%)
Saudi	411 (99%)
Hospital	
Um Alhamam Clinic	31 (7.5%)
Iskan Clinic	60 (14%)
Khashm Alaan Clinic	324 (78%)
BMI	
Healthy weight	27 (6.5%)
Obese	278 (67%)
Overweight	110 (27%)
Comorbidities	
Diabetes mellitus	152 (37%)
Dyslipidemia	136 (33%)
Hypertension	57 (14%)
Routine check-up	57 (14%)
Routine follow-up	41 (9.9%)
Osteoarthritis	19 (4.6%)
Vitamin D deficiency	17 (4.1%)
Cataract	15 (3.6%)
Urinary tract infection	10 (2.4%)

The median cholesterol level was 4.60 mmol/L (IQR 4.05-5.20). The median TG level at diagnosis was 2.90 mmol/L (IQR 2.63-3.34), and after treatment, it was reduced to 1.70 mmol/L (IQR 1.44-2.07).

Regarding statin usage, Rosuvastatin Ca (10 mg) was prescribed to 1 (0.2%) patient, Rosuvastatin (20 mg) to 3 (0.7%), Atorvastatin Ca (10 mg) to 12 (2.9%), Atorvastatin Ca (20 mg) to 30 (7.2%), Atorvastatin Ca (40 mg) to 7 (1.7%), Simvastatin (10 mg) to 2 (0.5%), Simvastatin (20 mg) to 4 (1.0%), and Simvastatin (40 mg) to 2 (0.5%). All these findings are summarized in Table 2.

**Table 2:** Lipid profile and statin use among study participants. ^1^Data are presented as median (IQR); n (%).

Characteristics	N = 415
Cholesterol (mmol/L)	4.60 (4.05-5.20)
Triglycerides at diagnosis (mmol/L)	2.90 (2.63-3.34)
Triglycerides after treatment (mmol/L)	1.70 (1.44-2.07)
Statin type and dosage	
Rosuvastatin Ca (10 mg)	1 (0.2%)
Rosuvastatin (20 mg)	3 (0.7%)
Atorvastatin Ca (10 mg)	12 (2.9%)
Atorvastatin Ca (20 mg)	30 (7.2%)
Atorvastatin Ca (40 mg)	7 (1.7%)
Simvastatin (10 mg)	2 (0.5%)
Simvastatin (20 mg)	4 (1.0%)
Simvastatin (40 mg)	2 (0.5%)

Age (p = 0.4), gender (p > 0.9), and body mass index (p = 0.8) were not associated with differences in TG levels. Marital status was significantly associated with differences in TG levels (p = 0.032), with a higher reduction observed among married participants compared to unmarried individuals.

Among medication users, Rosuvastatin (20 mg) (p = 0.5), Rosuvastatin Ca (10 mg) (p = 0.8), Atorvastatin Ca (20 mg) (p > 0.9), and Atorvastatin Ca (10 mg) (p = 0.6) were not significantly associated with differences in TG levels. Similarly, Atorvastatin Ca (40 mg) (p = 0.2), Simvastatin (20 mg) (p = 0.6), and Simvastatin (40 mg) (p = 0.9) were not associated with differences in TG levels. Lastly, Simvastatin (10 mg) showed a significant difference in TG level (p = 0.049). Although Simvastatin 10 mg demonstrated a statistically significant association with TG reduction (p = 0.049), this result is based on only two patients and should be interpreted with caution. All statistical associations are shown in Table 3.

**Table 3:** Association between differences in triglyceride levels, demographic characteristics, and medications used. ^1^Difference: median (IQR); ^2^Kruskal-Wallis rank-sum test; Wilcoxon rank-sum test.

Characteristics	N = 415^1^	p-value²
Age		0.4
38-48 years	1.30 (0.64-1.96)	
49-59 years	1.42 (0.89-1.92)	
60-70 years	1.19 (0.74-1.72)	
71 years and above	1.09 (0.75-1.71)	
Gender		>0.9
Female	1.24 (0.80-1.75)	
Male	1.20 (0.71-1.80)	
Marital status		0.032
Married	1.29 (0.84-1.79)	
Unmarried	1.03 (0.57-1.58)	
BMI		0.8
Healthy weight	1.37 (0.74-1.85)	
Obese	1.26 (0.85-1.79)	
Overweight	1.14 (0.63-1.76)	
Rosuvastatin 20 mg		0.5
No	1.24 (0.78-1.79)	
Yes	0.92 (0.84-1.23)	
Rosuvastatin Ca 10 mg		0.8
No	1.23 (0.77-1.79)	
Yes	1.39 (1.39-1.39)	
Atorvastatin Ca 20 mg		>0.9
No	1.26 (0.78-1.78)	
Yes	1.18 (0.82-2.00)	
Atorvastatin Ca 10 mg		0.6
No	1.21 (0.79-1.78)	
Yes	1.54 (0.64-2.05)	
Atorvastatin Ca 40 mg		0.2
No	1.25 (0.80-1.79)	
Yes	0.91 (0.53-1.26)	
Simvastatin 20 mg		0.6
No	1.24 (0.79-1.79)	
Yes	1.13 (0.52-1.60)	
Simvastatin 40 mg		0.9
No	1.24 (0.78-1.79)	
Yes	1.31 (0.60-2.01)	
Simvastatin 10 mg		0.049
No	1.21 (0.77-1.78)	
Yes	3.04 (2.58-3.51)	

**Figure 1:**
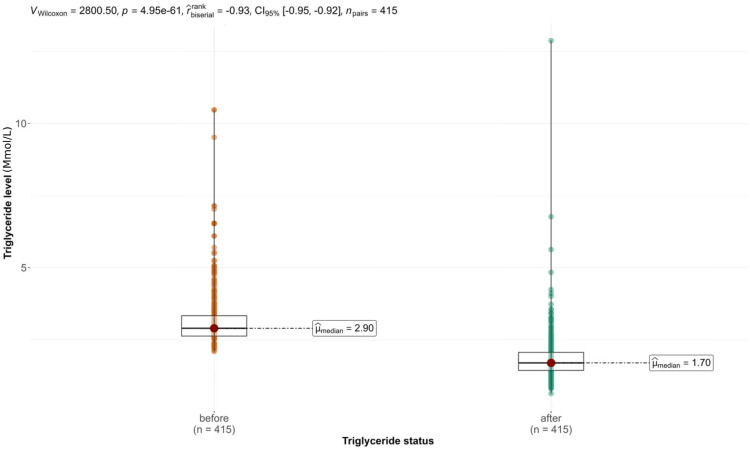
Level of control of triglycerides.

The median TG level before treatment was 2.90 mmol/L and was reduced to 1.70 mmol/L after treatment. The Wilcoxon signed-rank test indicated a significant reduction in TG levels, with a large negative effect size suggesting a substantial reduction after treatment (V = 2800.50, p < 0.001, effect size = -0.93). This change is demonstrated in Figure 1.

## Discussion

HTG is considered one of the most common lipid disorders worldwide and a significant risk factor, being specifically associated with acute pancreatitis (AP) and CVDs [8-9]. Globally, the prevalence of HTG varies across different populations, with studies reporting prevalence rates of 25.9% in the United States and approximately 27% in Spain [4-5]. Locally, in Saudi Arabia, the estimated prevalence rates range from 17% to as high as 40.3% in different studies [6-7].

This study primarily aimed to assess the level of control of HTG among patients attending Primary Health Care (PHC) clinics at the National Guard Health Affairs (NGHA) in Riyadh, Saudi Arabia. The study included 415 participants with a median age of 62 years, predominantly Saudi nationals, with the majority being female (59%). Overall, the results indicated a significant reduction in TG levels, from a median of 2.90 mmol/L at diagnosis to around 1.70 mmol/L after appropriate treatment and management (p < 0.001). These findings align with previous research emphasizing the importance of both pharmacological and non-pharmacological interventions in HTG management [10-11].

Consistent with international evidence, our study demonstrated significant improvement in TG levels through both pharmacological and lifestyle modification approaches. Notably, 67% of the patients included in the study were obese and 27% were overweight, highlighting the need for intensive weight-loss interventions. Obesity remains a well-established and critical risk factor for all forms of dyslipidemia, and weight reduction has been shown to be an essential strategy for significantly lowering TG levels [10-11]. Other lifestyle interventions, including dietary changes, increased physical activity, and smoking cessation, have also been shown to successfully reduce elevated TG levels [9]. However, our study did not assess adherence to lifestyle modification regimens, which remains an opportunity for future research.

Regarding pharmacological treatments, although it is well established that statins primarily target LDL-C levels, they have also been recognized for their role in reducing TG levels [9]. The nominal statistical significance observed for simvastatin 10 mg is limited by the very small number of users (n = 2) and should be considered exploratory, with a high potential for Type I error. Larger studies are needed to validate whether this signal has clinical relevance. In contrast, other statins, including rosuvastatin (10 mg and 20 mg) and atorvastatin (10 mg, 20 mg, and 40 mg), the doses available in NGHA, did not show any statistically significant effect. This finding is consistent with previously published literature indicating that different statin agents and dosages may have variable impacts on TG levels [10-11].

With regard to the association between demographic and clinical characteristics and TG reduction, our study did not find any significant association between TG level changes and age, gender, or BMI. Interestingly, this contrasts with some studies suggesting that higher BMI and increasing age are associated with poorer lipid control [9]. One possible explanation for this finding is the presence of other comorbidities that may influence lipid metabolism and response to treatment, such as diabetes and hypertension. The only notable finding was the significant association with marital status (p = 0.032), with married individuals experiencing greater reductions compared to their unmarried counterparts. Prior research has shown that social and psychological factors significantly influence both how consistently patients adhere to treatment plans and their overall health outcomes [9].

Despite our study’s strengths, there were several limitations. Firstly, this study was conducted exclusively in NGHA PHC Centers and may not adequately represent the entire Saudi population. Secondly, the data were primarily obtained through NGHA’s BESTCare electronic medical record system which, despite being comprehensive, has certain limitations in data collection, particularly regarding medication side effects, compliance, and adherence to lifestyle modifications, as these are not consistently documented. Thirdly, omega-3 fatty acids, which have been established to play a significant role in reducing TG levels [10-11], are not widely available in NGHA clinics and were therefore not assessed in our study. Furthermore, their unavailability may represent a potential gap in treatment options within this setting.

As for the clinical implications, our study highlights findings that should be addressed within primary healthcare settings. Firstly, given the high prevalence of obesity among participants, there is a critical need to implement more systematic and organized weight-management interventions in primary healthcare. Secondly, the significant association between marital status and TG reduction may suggest that social support and related psychosocial factors play a role in lipid management.

With these limitations in mind, further studies are recommended across multiple healthcare settings, incorporating a more diverse and larger sample size to achieve a comprehensive understanding of HTG control in Saudi Arabia. Additionally, non-pharmacological factors such as dietary modification, medication adherence, and exercise regimens should also be integrated and monitored closely in future studies, as they are crucial for understanding the full scope of TG management.

## Conclusions

In conclusion, our study offers important insights into the management of HTG among patients attending NGHA PHCs in Riyadh, Saudi Arabia. The findings indicate that the current treatment strategies are effective and consistent with international evidence. Furthermore, expanding treatment options and incorporating psychosocial support could enhance HTG management and help reduce the overall risk of cardiovascular and other metabolic diseases in Saudi Arabia.
